# Age-associated circadian period changes in Arabidopsis leaves

**DOI:** 10.1093/jxb/erw097

**Published:** 2016-03-24

**Authors:** Hyunmin Kim, Yumi Kim, Miji Yeom, Junhyun Lim, Hong Gil Nam

**Affiliations:** ^1^Department of Life Sciences, POSTECH, Hyojadong, Pohang, Gyeongbuk 37673, Republic of Korea; ^2^Department of New Biology, DGIST, Daegu 42988, Republic of Korea; ^3^Max-Planck Institute for Biology of Ageing, D-50931 Cologne, Germany; ^4^Integrative Biosciences & Biotechnology, POSTECH, Hyojadong, Pohang, Gyeongbuk 37673, Republic of Korea; ^5^Center for Plant Aging Research, Institute for Basic Science (IBS), Daegu 42988, Republic of Korea

**Keywords:** Arabidopsis, circadian clock, day length, leaf age, plant life history, *TOC1*.

## Abstract

As most organisms age, their appearance, physiology, and behaviour alters as part of a life history strategy that maximizes their fitness over their lifetime. The passage of time is measured by organisms and is used to modulate these age-related changes. Organisms have an endogenous time measurement system called the circadian clock. This endogenous clock regulates many physiological responses throughout the life history of organisms to enhance their fitness. However, little is known about the relation between ageing and the circadian clock in plants. Here, we investigate the association of leaf ageing with circadian rhythm changes to better understand the regulation of life-history strategy in Arabidopsis. The circadian periods of clock output genes were approximately 1h shorter in older leaves than younger leaves. The periods of the core clock genes were also consistently shorter in older leaves, indicating an effect of ageing on regulation of the circadian period. Shortening of the circadian period with leaf age occurred faster in plants grown under a long photoperiod compared with a short photoperiod. We screened for a regulatory gene that links ageing and the circadian clock among multiple clock gene mutants. Only mutants for the clock oscillator *TOC1* did not show a shortened circadian period during leaf ageing, suggesting that *TOC1* may link age to changes in the circadian clock period. Our findings suggest that age-related information is incorporated into the regulation of the circadian period and that *TOC1* is necessary for this integrative process.

## Highlight

The circadian period of the *Arabidopsis thaliana* leaf shortens with age. *TOC1* may be a critical signalling component linking the endogenous clock to leaf ageing pathways.

## Introduction

Almost all organisms undergo morphological and physiological changes as they age. Organisms possess signalling pathways that measure the passage of time and modulate the sequence of developmental change as part of a life history strategy to enhance fitness (Rougvie, 2001; Baurle and Dean, 2006). Ageing processes are genetically programmed in almost all higher organisms, from humans to plants (Lim *et al.*, 2007; Mitteldorf and Pepper, 2007). These organisms not only sense endogenous and exogenous signals for their survival but also predict future challenges, such as seasonal changes in climate and photoperiod. Thus, most multicellular organisms have evolved biological clocks consisting of multiple genes organized in feedback loops to adjust gene expression patterns and physiological processes to seasonal/environmental conditions.

The circadian clock is a part of the endogenous time measurement system in both plants and animals (Dunlap, 1999; Song *et al.*, 2015). Circadian clocks sense changes in environmental stimuli, such as light and temperature fluctuations, that follow day−night cycles, and can be entrained to generate internal rhythms of approximately 24h that are maintained independently of external stimuli (Millar, 2004; Harmer, 2009). The *Arabidopsis thaliana* circadian system consists of two major interconnected feedback loops, the morning and evening loops (Harmer, 2009; Pokhilko *et al.*, 2010; Pokhilko *et al.*, 2012). The morning loop includes the genes *CIRCADIAN CLOCK ASSOCIATED 1* (*CCA1*), *LATE ELONGATED HYPOCOTYL* (*LHY*), *PSEUDO-RESPONSE REGULATOR* (*PRR*) *7*, and *PRR9*, all of which show a peak of mRNA expression levels in the morning (Farre *et al.*, 2005; Mizuno and Nakamichi, 2005; Zeilinger *et al.*, 2006; Nakamichi *et al.*, 2010). The evening loop includes *TIMING OF CAB EXPRESSION 1* (*TOC1*), *GIGANTEA* (*GI*), *EARLY FLOWERING* (*ELF*) *3*, *ELF4*, and *LUX ARRYTHMO* (*LUX*), all of which show highest expression in the evening and are transcriptionally or translationally linked to the morning loop (Fowler *et al.*, 1999; Park *et al.*, 1999; McWatters *et al.*, 2000; Strayer *et al.*, 2000; Hazen *et al.*, 2005; Kolmos *et al.*, 2009; Kim *et al.*, 2012; Kim *et al.*, 2013). The orchestrated action of the oscillator components leads to the rhythmic behaviour of circadian outputs (Schaffer *et al.*, 1998; Wang and Tobin, 1998; Park *et al.*, 1999; Strayer *et al.*, 2000; Kolmos *et al.*, 2009; Kim *et al.*, 2013). The endogenous circadian clock of plants regulates many aspects of plant development over the life cycle, including chloroplast movement, stomatal opening, seedling growth, leaf movement, petal opening, and flowering (Nozue *et al.*, 2007; Sawa *et al.*, 2007; Haydon *et al.*, 2013). In contrast to the highly integrated circadian networks in mammals, plant rhythms appear to be less tightly coupled among cells, tissues, and organs (Thain *et al.*, 2002). This feature allows the individual plant organs to entrain to environmental signals independently (Thain *et al.*, 2000). Also, the same tissues at different locations within a plant (e.g., leaves) can individually modulate circadian periodicity according to unique conditions such as sun exposure (Thain *et al.*, 2000). However, the recent finding that a vascular clock can regulate flowering time suggests that at least one of the tissue-specific clocks in the plant can affect other physiological responses (Endo *et al.*, 2014). Further, the circadian clock in the shoot apex can function similarly to the animal master clock of the suprachiasmatic nucleus to synchronize the root circadian rhythm (Takahashi *et al.*, 2015).

Like other plant organs, many morphological and physiological changes occur in the leaf. Rosette leaves emerge from leaf primordia of the shoot apical meristem, expand laterally and distally, and differentiate with age (Efroni *et al.*, 2008; Bar and Ori, 2014). Many vital functions of plants take place in the leaves, such as photosynthesis, photorespiration, and transpiration. Importantly, leaves act as a sink organ for storing organic compounds during growth and maturation. Flower-inducing hormone, so called florigen, is also synthesized in leaves in response to environmental stimuli such as photoperiod and temperature, and translocates into the shoot apical meristem (Tsukaya, 2013). Then, leaves become active source organs to transfer carbon material into the seeds before eventual senescence.

In this study, we examined the relation between leaf ageing and the circadian clock in Arabidopsis leaves. We found that the circadian period differed among leaves within a single plant. We observed the circadian period shortening with leaf ageing by measuring the promoter activity and the expression of circadian clock genes. Changes in the circadian period with leaf age occurred faster in plants grown under long day conditions than under short day conditions. Further, *TOC1* gene mutants showed no such age-dependent changes, suggesting that the circadian rhythm is regulated by age through the *TOC1*, clock oscillator.

## Materials and Methods

### Plant material

To monitor changes in clock gene expression with age and identify leaf age-dependent circadian regulators, we generated several transgenic *Arabidopsis thaliana* lines expressing the firefly luciferase gene under control of the clock responsive *COLD CIRCADIAN RHYTHM AND RNA BINDING 2* (*CCR2*), and *CCA1* promoters. Before the cross, *cca1-11* (on the Ws background) (Gould *et al.*, 2006) and *toc1-1* (on C24) (Millar *et al.*, 1995) were backcrossed three times with Col-0 wild type. We then crossed *CCR2p*::*LUC* with *cca1-11*, *toc1-1*, and *toc1-101* mutants (Kikis *et al.*, 2005) and *CCA1p::LUC* with *lhy-20* mutants (Michael *et al.*, 2003) to measure circadian rhythmicity. *CCA1p::LUC* was introduced into the *prr7-3* and *prr9-1* mutants by *Agrobacterium* transformation.

### Plant growth conditions

**Fig. 1.**
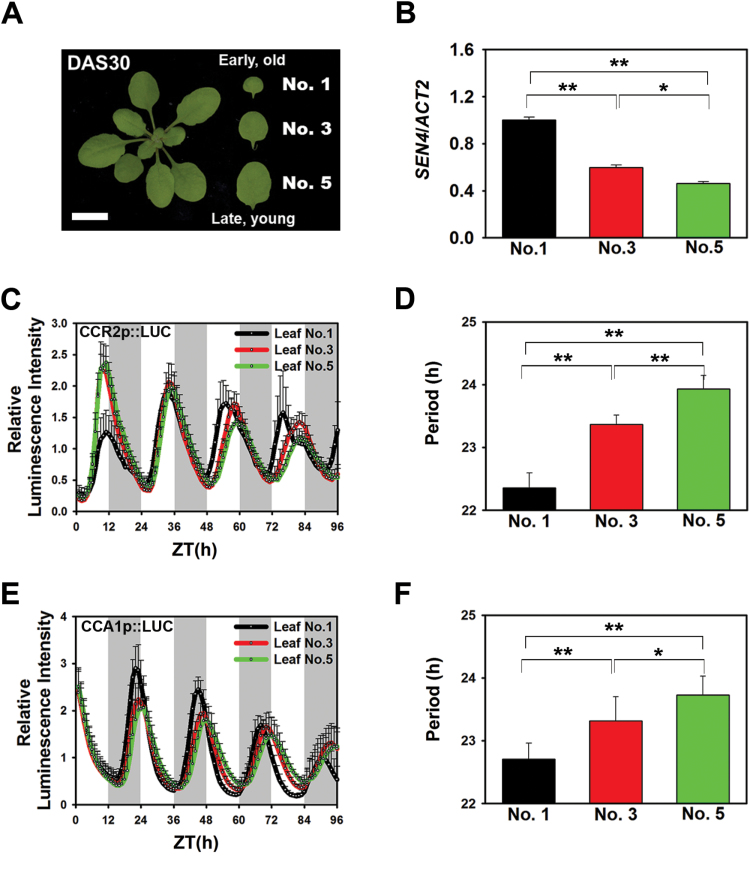
Early emerged (older) Arabidopsis leaves show a shorter circadian period than late emerged (younger) leaves.

*Arabidopsis thaliana* was grown in an environmentally controlled growth room at 22 °C under a 12-h light–12-h dark cycle (12L/12D), a 16-h light–8-h dark cycle (16L/8D; longer photoperiod), or an 8-h light–16-h dark cycle (8L/16D; shorter photoperiod) using 100 μmol m^−2^ s^−1^ white light. The plants were then transferred to continuous white light at the same light intensity to measure rhythmic changes in luciferase emission from transgenic leaves. All experiments except that shown in Fig. 1 were performed using the third and fourth rosette leaves.

(A) A wild type *Arabidopsis thaliana* rosette leaf at 30 days after sowing (DAS) showing early emerged (older) and later emerged (younger) leaves. Scale bar: 1cm. (B) Expression of the age-induced marker gene *SEN4* at the indicated leaf number. The first, third, and fifth leaf samples were harvested at zeitgeber (ZT) 4. Data are presented as the mean ± standard error (SE) of biological triplicates. (C, E) Time course of bioluminescence levels in plants expressing *CCR2p::LUC* (C) or *CCA1p::LUC* (E). Luminescence intensities were measured every hour under continuous light (LL) conditions starting at the leaf number indicated. (D, F) Circadian period estimates of the activities of *CCR2* (D) and *CCA1* (F) promoters. Data are presented as the mean ± standard deviation (SD) from eight leaves. The single (*P*<0.05) and double (*P*<0.01) asterisks indicate significant difference (one-way ANOVA followed by Tukey’s HSD test for pairwise comparisons). White bars indicate subjective day, and gray shading indicates subjective night. (This figure is available in colour at *JXB* online.)

### Measurement of mRNA expression levels

Total mRNA was extracted from the leaves using WelPrep (Welgene, Daegu, Korea). Contaminating DNA was removed by digestion with DNase I (Ambion, Austin, TX, USA). For each sample, 0.75 μg of total mRNA was reverse-transcribed using ImProm II reverse transcriptase (Promega, Madison, WI, USA). The quantity of each transcript in a sample was measured using real-time PCR with SYBR Premix Ex Taq (Takara, Shuzo, Kyoto, Japan) and an ABI 7300 real-time PCR system (Applied Biosystems, Foster City, CA, USA). The primers used in this study and their sequences are listed in Supplementary Table S1 at *JXB* online.

### Luminescence assay

Transgenic plants expressing luciferase under the control of the *CCR2* and *CCA1* promoters (Strayer *et al.*, 2000) were used in this assay. The third and fourth rosette leaves were excised at their petioles from transgenic plants and transferred to 24-well microplates containing 500 µM luciferin (SYNCHEM, Felsberg/Altenburg, Germany). Luminescence images were acquired every hour for 4 days and luminescence intensities from each leaf were imported into the Biological Rhythms Analysis Software System (BRASS) (Southern and Millar, 2005). Circadian period lengths were calculated using the FFT-NLLS suite (Plautz *et al.*, 1997).

## Results

### Circadian period heterogeneity of leaves within a single Arabidopsis plant

Arabidopsis leaves are sequentially generated as the plant ages. A leaf that emerges earlier is older than a leaf that emerges later; thus, leaves of various ages occur in a single plant (Zentgraf *et al.*, 2004). We first analysed whether circadian rhythms are synchronized among leaves within a plant. The circadian rhythms of the first, third, and fifth emerged leaves were examined at 30 days after sowing (DAS) (Fig. 1A). Expression of the age-associated marker *SENESCENSE 4* (*SEN4*) was higher in the earlier emerged leaf (leaf number 1) than in the later emerged leaf (leaf number 5), which is consistent with a previous report (Fig. 1B) (Zentgraf *et al.*, 2004). Cyclic activities of the *CCR2* (clock output) and *CCA1* (core oscillator) gene promoters were measured at 30 DAS in the leaves of transgenic plants expressing *CCR2p::Luciferase* (*LUC*) and *CCA1p::LUC*, respectively (Strayer *et al.*, 2000). Both transgenic plants were entrained under a 12L/12D cycle. Then, leaves were transferred to continuous light (LL) conditions to examine the endogenous circadian rhythm. The rhythmic expression levels were robust in both transgenic plants and varied with the leaf number. Specifically, the circadian periods of these reporters were shorter in early-emerged leaves, approximately 22.6h in the first emerged leaves versus nearly 24h in the fifth emerged leaves (Fig. 1D, F). Thus, the circadian clock period length varies among leaves of a single plant according to time of leaf emergence. This heterogeneity is consistent with previous reports that plant circadian rhythms are often uncoupled among cells and tissues (Wenden *et al.*, 2012; Endo *et al.*, 2014). Interestingly, this also implies a possibility that the circadian rhythm might be correlated with leaf age. Thus, we hypothesized that there is an age-dependent circadian regulation in Arabidopsis leaves. To test this hypothesis, we focused on the third and fourth leaves of an Arabidopsis rosette in order to be certain of the leaf age and to avoid mixing leaf ages within a single plant (Zentgraf *et al.*, 2004).

### The circadian period is shortened with leaf ageing

**Fig. 2.**
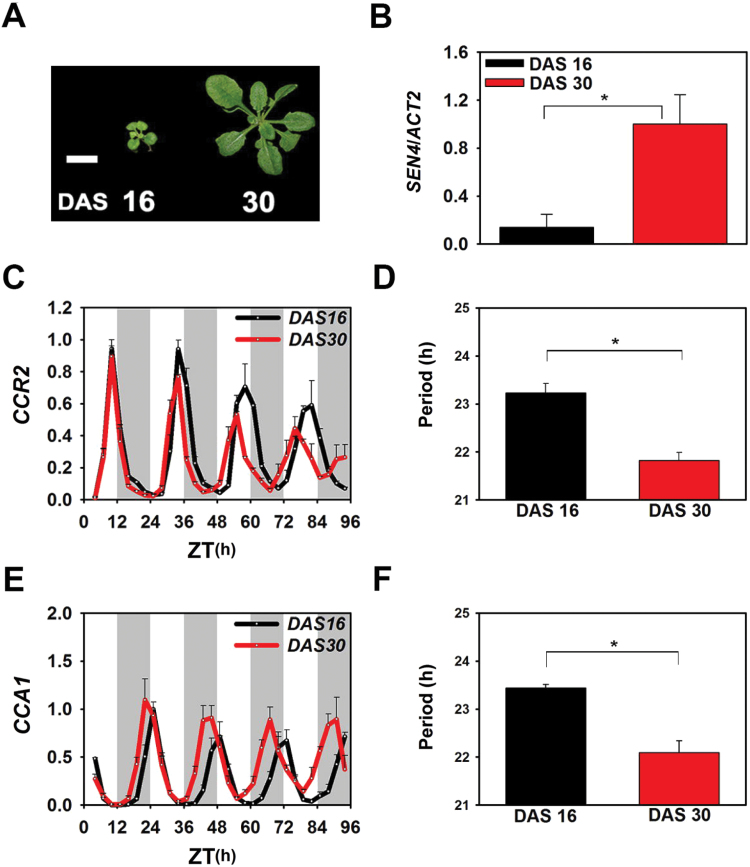
Circadian period is getting shorter with Arabidopsis leaf age.

To address how circadian rhythms respond chronologically, changes in the circadian rhythm were examined as the plant leaf ages. Experiments were performed before flowering to avoid the possible confounding effects of flowering (Hayama and Coupland, 2003). We harvested the third and fourth leaves from plants at 16 and 30 DAS for 4 days under free-running cycles entrained by a 12L/12D cycle (Fig. 2A). To objectively measure leaf age, we introduced *SEN4* as a molecular marker and measured *SEN4* mRNA expression in 16 and 30 DAS at 4h after lights on [zeitgeber (ZT) 4] (Oh *et al.*, 1996; Gan and Amasino, 1997). *SEN4* expression in leaves increased approximately 10-fold from 16 to 30 DAS, indicating that the leaves under investigation were aged (Fig. 2B). We measured the *CCR2* gene at these stages and found that the cycling of *CCR2* gene expression was robust at both 16 and 30 DAS, but that the circadian period was significantly shorter at 30 DAS compared with 16 DAS (Fig. 2C, D). Consistent with a shorter circadian period with age, the phases of the circadian peak were advanced at 30 DAS relative to 16 DAS for the third and fourth emerged leaves (Fig. 2C, D).

(A) Images show WT plants at the indicated leaf ages. Scale bar: 1cm. (B) Expression of the age-induced marker gene *SEN4* at the indicated leaf ages. Leaf samples were collected at ZT 4. (C) Expression of *CCR2*, a clock output gene, under LL. (E) Expression of *CCA1*, a clock oscillator gene, under LL. (D, F) Circadian period estimates for *CCR2* (D) and *CCA1* (F). Data are presented as the mean±SE of biological triplicates. The third and fourth leaf samples were collected for this analysis. mRNA levels were measured using quantitative RT-PCR and then normalized to *ACT2* expression. The asterisk indicates that the period values differ significantly (*P*<0.05) from young leaves (Tukey’s HSD test after one-way ANOVA). White bars indicate subjective day, and gray shading indicates subjective night. (This figure is available in colour at *JXB* online.)

Next, we examined whether the shorter period of circadian output genes such as *CCR2* in aged leaves results from parallel changes in the central oscillator by measuring the cyclic expression of nine core oscillator genes (Supplementary Fig. S1A). All monitored genes showed significantly shortened circadian periods in aged leaves compared with young leaves (~1h difference), thus closely recapitulating the change in cycling behaviour of *CCR2* expression with age (Fig. 2F and Supplementary Fig. S1B–I). We also tested whether the phase advance of expression of the core oscillator genes can be seen under diurnal conditions. However, the phases of the oscillator genes were not significantly altered from young to aged leaves (Supplementary Fig. S2). These parallel changes in circadian periods of central clock genes under free-running circadian cycles suggest age-dependent changes in multiple periodic physiological processes under control of the circadian core oscillators.

We further confirmed this circadian period shortening with leaf age using transgenic plants carrying *CCR2p*::*LUC* and *CCA1p*::*LUC* (Supplementary Fig. S3). Both transgenic plants were entrained under 12L/12D cycles, and the rhythmic expression levels of these reporter genes were measured in detached third and fourth leaves under continuous light. Similar to *CCR2* and *CCA1* genes in attached leaves, the circadian periods of *CCR2* and *CCA1* promoter activity were significantly shorter at 30 DAS (again by approximately 1h) compared with 16 DAS (Supplementary Fig. S3). Collectively, these results suggest that the circadian periods of both core oscillator and clock output genes progressively decrease with leaf age.

### The age-dependent change in circadian period is accelerated under a longer photoperiod

**Fig. 3.**
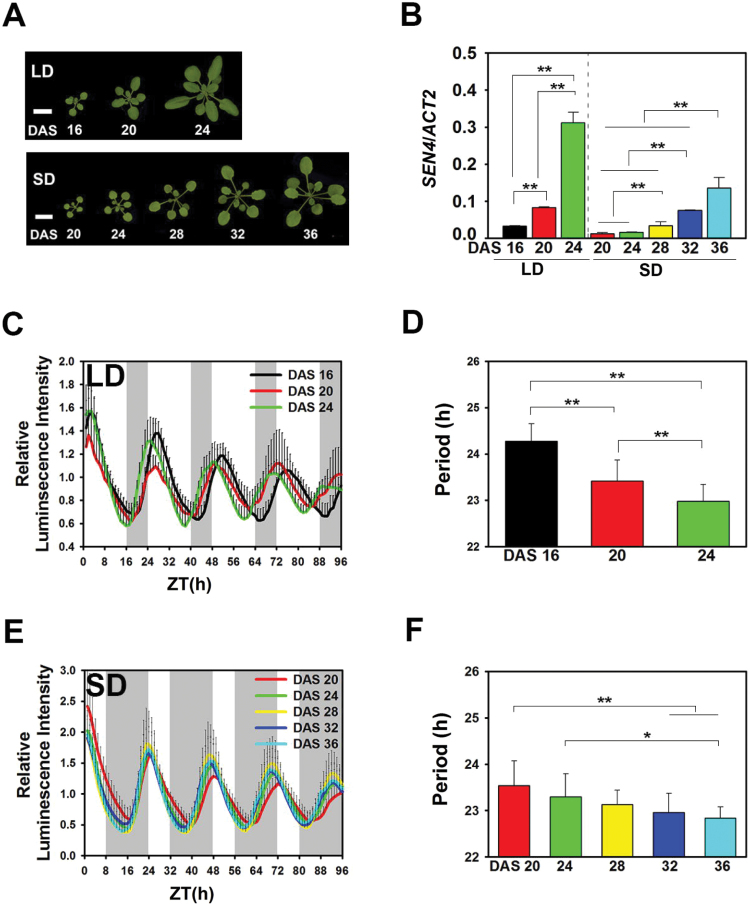
Arabidopsis leaves show accelerated circadian period shortening under a long photoperiod

*Arabidopsis thaliana* is a facultative long-day plant, indicating that the transition from the vegetative to reproductive stage is faster under a long photoperiod than under a short photoperiod (Corbesier *et al.*, 1996). Sensing the external photoperiod is one mechanism through which the endogenous circadian clock controls flowering in Arabidopsis. Thus, we hypothesized that the rate of circadian period shortening during leaf ageing would vary with day length. To test this hypothesis, the cyclic luminescence activity of the *CCA1* promoter was measured in plants grown under short day (SD) (8L/16D) and long day (LD) (16L/8D) conditions. Leaves were collected before flowering every 4 days, starting at 20 DAS for plants grown under SD conditions and at 16 DAS for plants grown under LD conditions (Fig. 3A). *SEN4* expression progressively increased under both photoperiod conditions, but the rate of increase was higher under the LD than under SD conditions, indicating that leaf ageing is faster under a long photoperiod compared with a short photoperiod (Fig. 3B). The circadian period gradually shortened with leaf age under both conditions (Fig. 3D, F). The circadian period under SD was significantly shortened at 32 and 36 DAS compared with the period at 20 DAS (Fig. 3F). The period shortening under SD took much longer than under LD, which correlates with the milder increase of *SEN4* expression under SD compared with LD (Fig. 3B). This result indicates that leaf ageing differs with the day length and that this correlates to the shortening of the circadian period with leaf ageing.

. (A) Images of plants grown under long and short photoperiod conditions at the indicated age. Scale bar: 1cm. (B) Expression of the age-induced marker gene *SEN4* at the indicated leaf ages. The third and fourth leaf samples were collected at ZT 4. Data are presented as the mean±SE of biological triplicates. (C, E) *CCA1* promoter activity was measured by monitoring the luminescence intensity from leaves of transgenic plants expressing luciferase under the control of the *CCA1* promoter. Luminescence intensities were measured every hour at the indicated leaf age under LL conditions. (D, F) Circadian period estimates for *CCA1p::LUC* from data shown in (C, E). Data are presented as the mean±SD from 20 leaves. The single (*P*<0.05) and double (*P*<0.01) asterisks indicate significant difference (Tukey’s HSD test after one-way ANOVA). (This figure is available in colour at *JXB* online.)

### *TOC1* is involved in age-dependent changes in the circadian rhythm

**Fig. 4.**
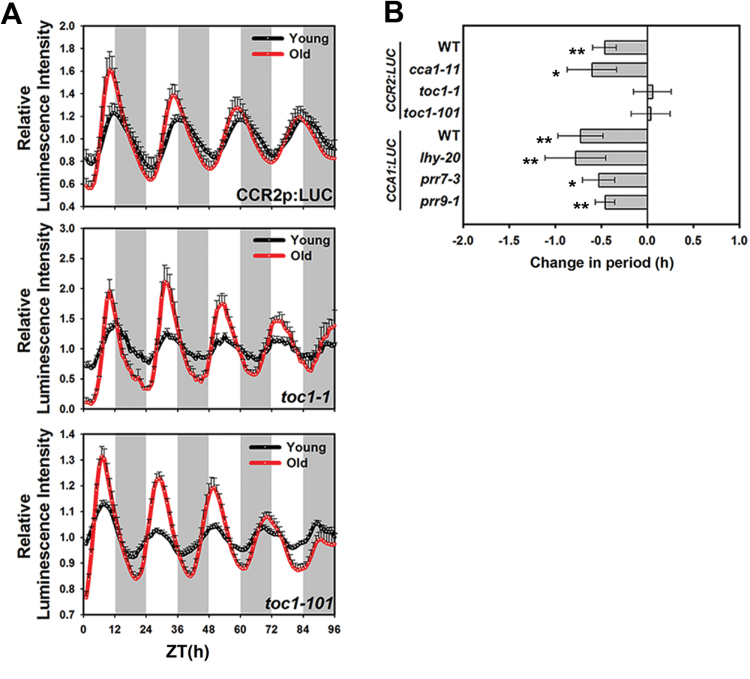
*TOC1* is a critical clock oscillator in the age-interacting clock network.

Leaf age affects the endogenous clock at the level of the core oscillator as well as at the level of clock output genes (Fig. 2 and Supplementary Fig. S1), suggesting that a component of the core oscillator acts to link leaf age to downstream effects on clock outputs. We screened several clock mutants that have defects in clock regulation during leaf ageing. Leaves from mutant and wild type (WT) plants grown under 12L/12D were collected at 18 and 28 DAS and the clock activities measured under continuous light. Consistent with the previous results, leaf age significantly shortened the circadian periods of *CCR2p::LUC* and *CCA1p::LUC* (by approximately 30min) in WT leaves (Fig. 4B). Similarly, the circadian periods significantly shortened with leaf age in *cca1-11*, *lhy-20*, *prr7-3*, and *prr9-1* mutants, and the differences in period between young and old leaves were statistically indistinguishable from WT plants (Fig. 4B and Supplementary Fig. S4). Interestingly, the circadian period in *toc1* mutants (*toc1-1* and *toc1-101*) did not shorten with leaf ageing in contrast to other clock mutants that we tested (Fig. 4B and Supplementary Fig. S4) and the circadian phase in *toc1* mutants was not advanced with leaf age (Fig. 4A). This finding implicates *TOC1* as a key regulator linking leaf ageing with changes in the endogenous circadian clock period.

(A) *CCR2* promoter activity was measured by monitoring luminescence intensity from the leaves of transgenic plants expressing luciferase under the control of the *CCR2* promoter. Luminescence intensities were measured every hour under LL conditions. White bars indicate day, and grey bars indicate night. (B) Change in the circadian period of *CCR2p::LUC* and *CCA1p::LUC* activity in several clock mutants. Grey bars indicate the circadian period change between 18 DAS and 28 DAS. Data are presented as the mean±SD of about 16 third and fourth leaves. The single (*P*<0.05) and double (*P*<0.01) asterisks indicate significant difference from the period of young leaves (Tukey’s HSD test after one-way ANOVA). (This figure is available in colour at *JXB* online.)

## Discussion

We found that each leaf in an *Arabidopsis thaliana* plant has a different circadian period depending on its age. The older, early emerged leaves of the Arabidopsis rosette had a shorter circadian period than the younger, later emerged leaves (Fig. 1). This finding indicates that the circadian rhythm is not synchronized within a plant. In the mammalian circadian system, the suprachiasmatic nucleus generates a ‘master’ circadian rhythm that modulates peripheral clocks to synchronize whole-body circadian rhythms (Reppert and Weaver, 2002). In contrast, plants have an independent autonomous circadian system at the cellular and tissue levels (Thain *et al.*, 2000), which could allow differential responses to similar environmental conditions. This suggests that the circadian rhythm in a leaf is spatially distinguishable, and thus, it might individually respond to age (Fig. 1).

Changes in the cyclic behaviour of the core circadian system regulates numerous developmental outputs throughout the plant life cycle, including photoperiodic control of seedling growth in young stages and photoperiodic control of flowering in mature stages (Nozue *et al.*, 2007; Sawa *et al.*, 2007; de Montaigu *et al.*, 2010; McWatters and Devlin, 2011; Kim *et al.*, 2012). In Arabidopsis, as leaves age, a globally orchestrated change was observed in the circadian periods of core oscillators (Fig. 2 and Supplementary Fig. S1). Changes in the rhythmic behaviour of even a single component of the core circadian oscillator may affect diverse aspects of plant physiology (Schaffer *et al.*, 1998; Wang and Tobin, 1998; Park *et al.*, 1999; Strayer *et al.*, 2000; Doyle *et al.*, 2002). It is thus conceivable that age-dependent changes in the circadian rhythm provide a regulatory means of linking age-related information to downstream developmental events.

Many physiological processes are dependent on day length, such as flowering and leaf senescence (Corbesier *et al.*, 1996; Nooden *et al.*, 1996). Arabidopsis developmental processes are induced more rapidly under long day than under short day conditions. Our results suggest that the shortening of the circadian period is age dependent and responds to the photoperiod. The circadian period shortens rapidly with leaf age under long photoperiod compared with short photoperiod conditions (Fig. 3). This finding suggests that the circadian clock and ageing and environmental signals work interactively in Arabidopsis developmental processes.

*TOC1* is one of the clock oscillators in the Arabidopsis circadian network. *TOC1*-deficient mutants exhibit a short-period phenotype in the seedling stage and an early flowering phenotype in the mature stage (Somers *et al.*, 1998; Strayer *et al.*, 2000). TOC1 also functions in photomorphogenic processes (Mas *et al.*, 2003). We found that leaf circadian periods in *toc1* mutants (*toc1*-*1* and *toc1*-*101*) were insensitive to leaf ageing (Fig. 4). TOC1 is closely associated with the abscisic acid (ABA) signalling pathway. ABA is a phytohormone that acts to coordinate stress responses to various stressor combinations. In addition, ABA is known to increase with leaf age and to regulate some features of leaf development (Breeze *et al.*, 2011; Lee *et al.*, 2011). ABA induces *TOC1* mRNA expression through *ABA BINDING PROTEIN* (*ABAR*) (Legnaioli *et al.*, 2009). Reciprocally, the circadian clock affects the oscillations of several ABA signalling genes, including *ABI1*, *RCAR1*, and *ABF3* (Seung *et al.*, 2012). However, ABA treatment of seedlings lengthens the circadian period under continuous light conditions (Hanano *et al.*, 2006). The functional interactions between ABA signalling and TOC1 during leaf ageing are still largely unknown. However, given that ABA does regulate *TOC1* expression, it is a potential candidate age-related stimulus affecting the circadian clock through TOC1.

It remains unclear how ageing is associated with changes in the circadian system, particularly whether there is indeed a causal relationship between them or if such observations arise merely from coincidence. It is not yet known how age-dependent changes in the circadian clock system and infradian developmental events such as flowering and senescence are interlinked. Our results described here may provide the first insights for understanding how leaf age and age-dependent changes in the circadian clock are incorporated into age-dependent developmental decisions.

## Supplementary data

Supplementary data are available at *JXB* online.

Figure S1. The rhythmic behaviour of core clock oscillators differs between young and aged leaves.

Figure S2. The phase of the clock oscillator genes is not significantly different in young and aged leaves under diurnal condition.

Figure S3. The rhythmic behaviour of clock gene promoters differs between young and aged detached leaves.

Figure S4. Age-dependent circadian rhythms in several clock oscillator mutants.

Table S1. Oligonucleotides used for real-time PCR.

Supplementary Data
