# Supplementary material for: Age-associated circadian period changes in Arabidopsis leaves
Source: J Exp Bot. 2016 Mar 24;67(9):2665–73. doi: 10.1093/jxb/erw097 (PMC4861015; doi:10.1093/jxb/erw097)
Supplement: Supplementary Data [file supp_67_9_2665__index.html]

Age-associated circadian period changes in Arabidopsis leaves — Age-associated circadian period changes in Arabidopsis leaves — Supplementary Data 

# Age-associated circadian period changes in Arabidopsis leaves

## Supplementary Data

Data files

- supplementary\_figures\_S1\_S4\_table\_S1.pdf - Supplementary Data
