# Supplementary material for: Age-associated circadian period changes in Arabidopsis leaves
Source: J Exp Bot. 2016 Mar 24;67(9):2665–73. doi: 10.1093/jxb/erw097 (PMC4861015; doi:10.1093/jxb/erw097)
Supplement: Supplementary Data [file supp_erw097_supplementary_figures_S1_S4_table_S1.pdf]

Supplementary Data

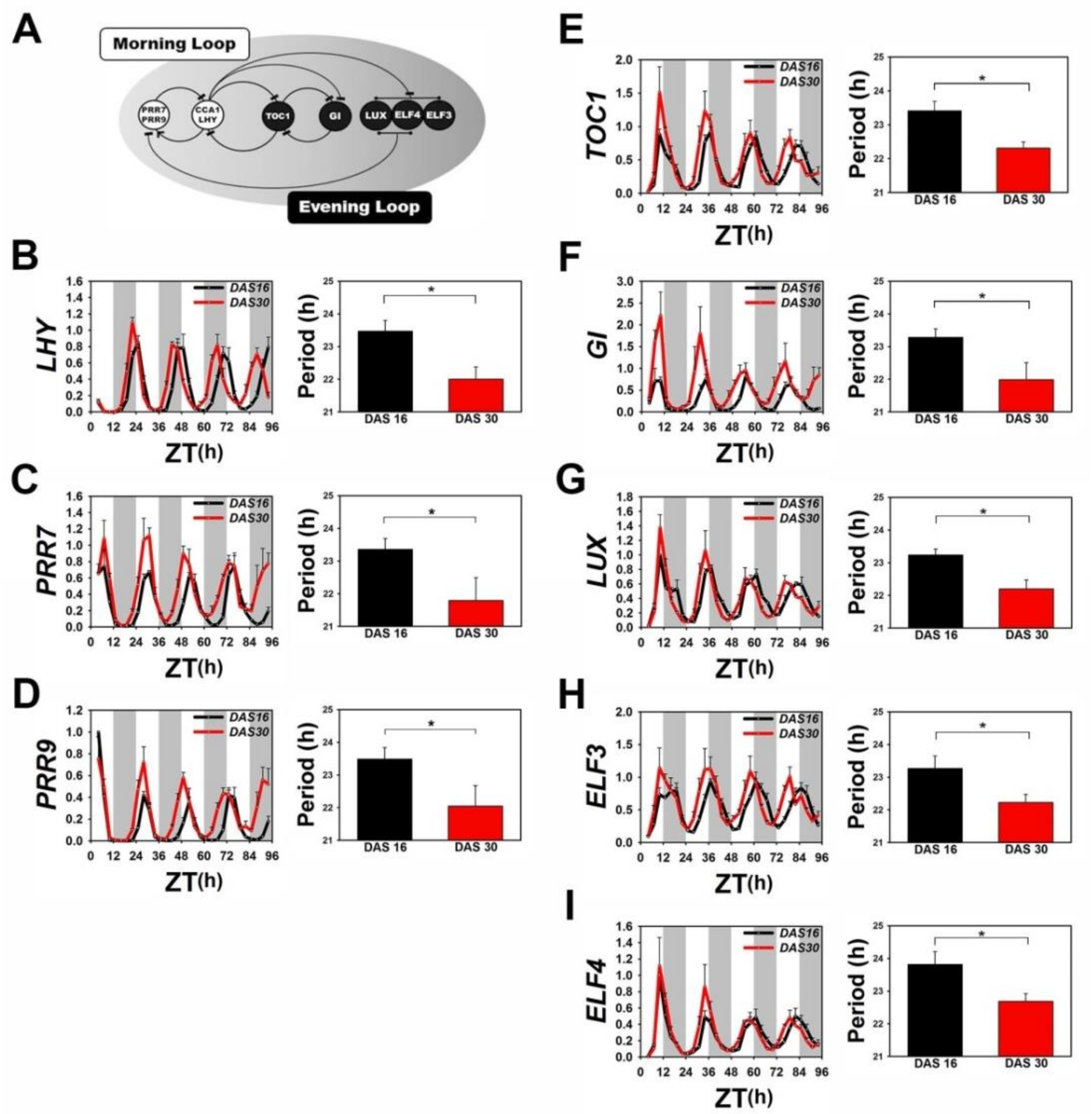

Fig. S1. The rhythmic behavior of core clock oscillators differs between young and aged leaves

(A) Model of the *Arabidopsis* circadian clock. (B–D) Time courses of the expression of genes from the morning loop of the circadian oscillator under LL. (E–I) Time courses of the expression of genes from the evening loop of the oscillator under LL. The right-hand panels show the mean period for each gene. Data are presented as the mean ± SE of biological triplicates. mRNA levels were measured using

quantitative RT-PCR and then normalized using the values of *ACT2*. The single asterisk indicates that the period values differ significantly ($p < 0.05$) from those in young leaves (Tukey's HSD test after one-way ANOVA). Experiment was performed using the third and fourth rosette leaves. White bars indicate subjective day and gray shading indicates subjective night.

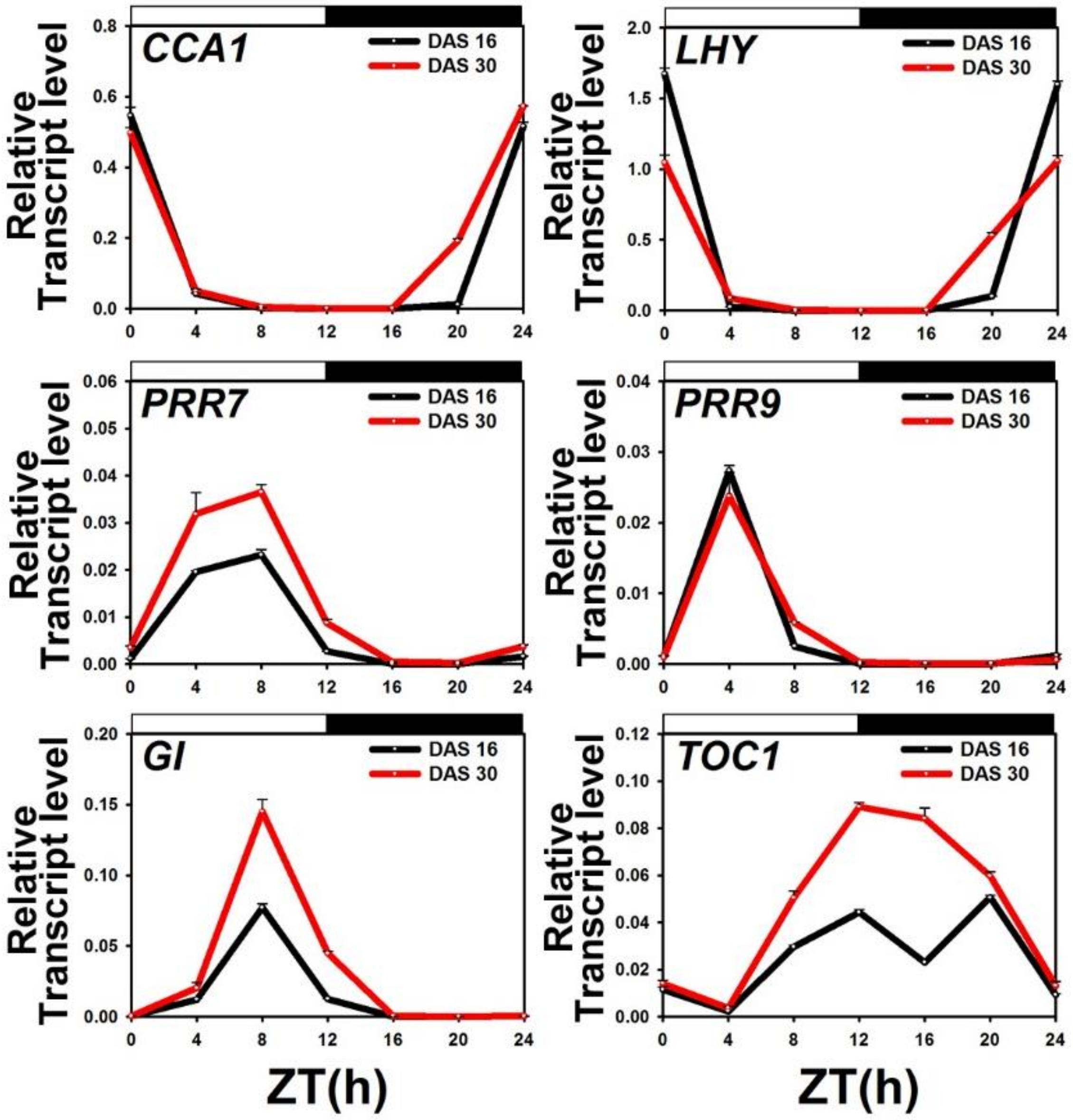

Fig. S2. The phase of the clock oscillator genes is not significantly different in young and aged leaves under diurnal condition.

Cycling expressions of several core genes in the circadian oscillator were measured at the indicated leaf age under diurnal condition (12-h light/ 12-h dark). Data are presented as the mean ± SE of biological triplicates mRNA levels were measured using semi-quantitative RT-PCR and then normalized using the values of *ACT2*. White and black bars indicate day and night, respectively.

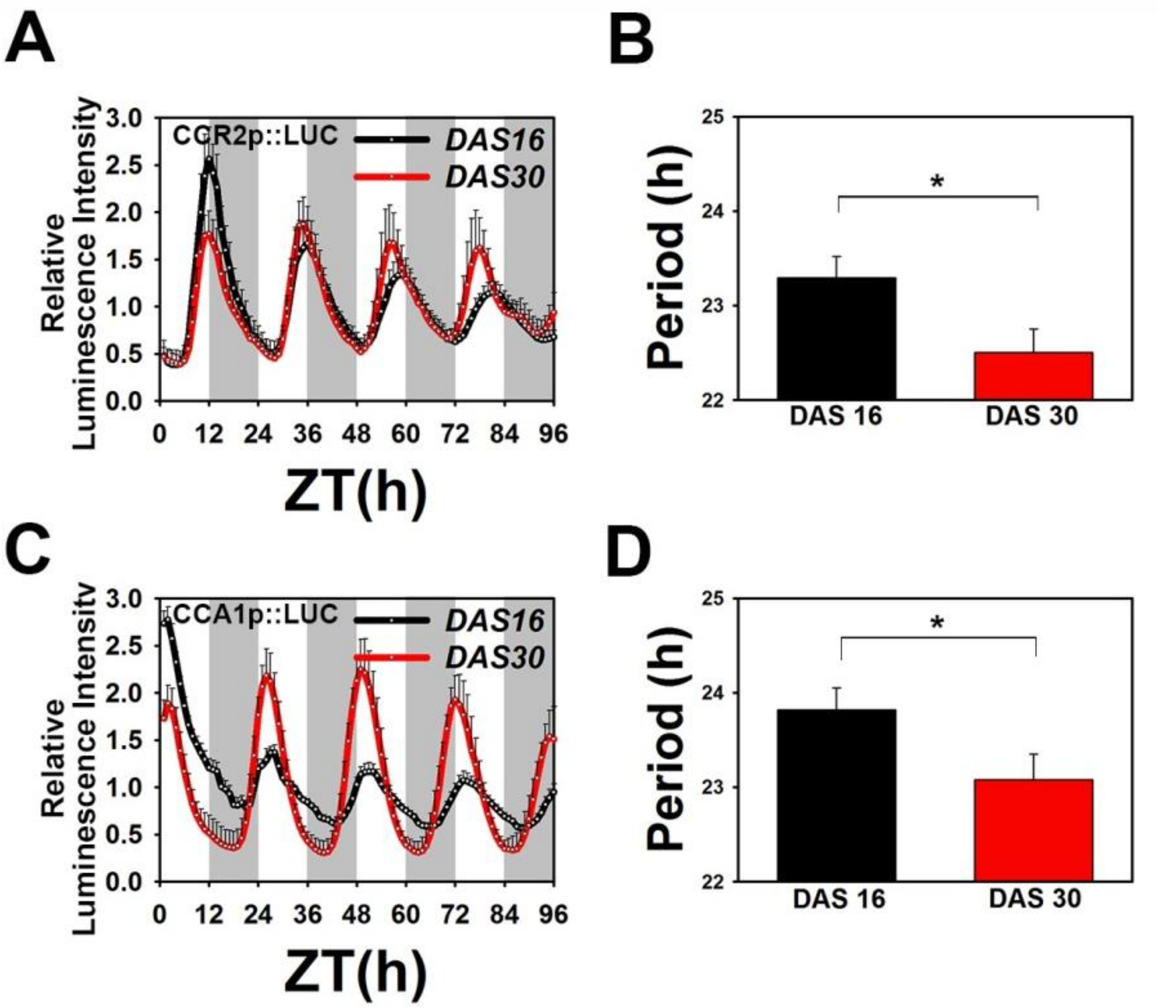

Fig. S3. The rhythmic behavior of clock gene promoters differs between young and aged detached leaves

Time course of bioluminescence levels in plants expressing *CCR2p*::*LUC* (A) or *CCA1p*::*LUC* (C). Luminescence intensities were measured every hour under LL conditions at the indicated leaf age under LL conditions. (B) Circadian period estimates for *CCR2p*::*LUC* from A data. (C) Circadian period estimates for *CCA1p*::*LUC* from C data. Data are presented as the mean ± SD from 12 leaves. The single ($p < 0.01$) asterisks indicate significant difference (Tukey's HSD test after one-way ANOVA). Experiment was performed using the third and fourth rosette leaves. White bars indicate subjective day and gray shading indicates subjective night.

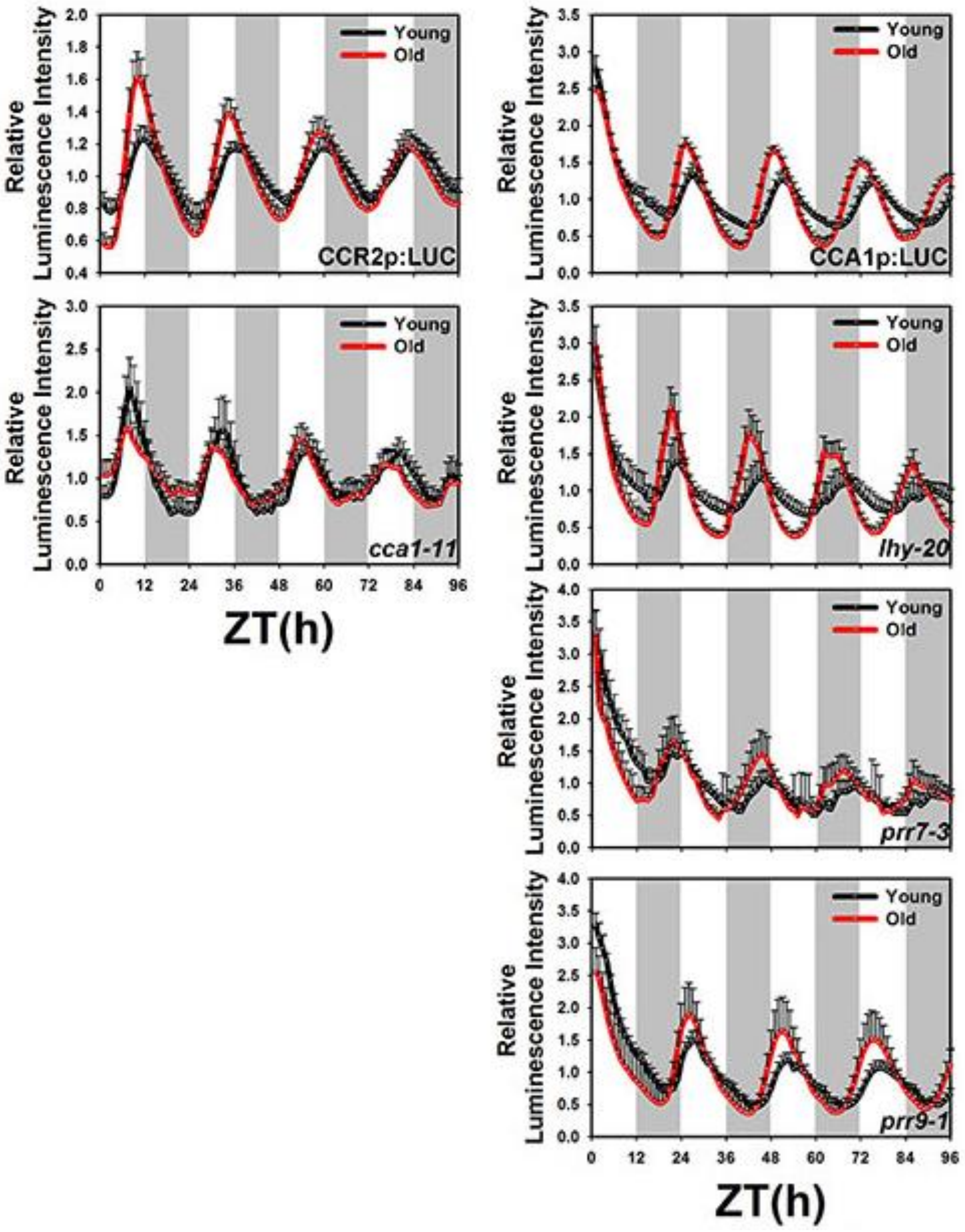

Fig. S4. Age-dependent circadian rhythms in several clock oscillator mutants. Time course of bioluminescence levels in plants expressing *CCR2p or CCA1p*::*LUC*. The promoter activity was measured by monitoring the luminescence intensity in transgenic plants expressing luciferase under the control of the *CCR2* or *CCA1* promoter. Luminescence intensities were measured every hour under continuous light (LL) conditions starting at the indicated leaf age. Luminescence intensities at each age were normalized to the mean intensity. Data are presented as the mean ± SD of 12 leaves. Experiment was performed using the third and fourth rosette leaves.

Table S1. Oligonucleotides used for real-time PCR.

Gene	AGI code	Sequence (5′ → 3′)	
		Forward	Reverse
CCR2	AT2G21660	CTTGATCTTCCAGTCTCACGAT	CGACGTTATTGATTCCAAGATCA
CCA1	AT2G46830	CAGCTCCAATATAACCGATCCAT	CAATTCGACCCTCGTCAGACA
LHY	AT1G01060	CAACAGCAACAACAATGCAACTAC	AGAGAGCCTGAAACGCTATACGA
PRR7	AT5G02810	GAATGTGCTGAGGCGTTCAGA	GGCTGGATTATACCTTGAGAAAGC
PRR9	AT2G46790	GTTGAAGAGGAAAGATCGATGCTT	CTGCTCTGGTACCGAACCTTTT
GI	AT1G22770	AATTCAGCACGCGCCTATTG	GTTGCTTCTGCTGCAGGAACTT
TOC1	AT5G61380	AATAGTAATCCAGCGCAATTTTCTTC	CTTCAATCTACTTTTCTTCGGTGCT
ELF3	AT3G21320	ATTGCTGCATCACCGGATCT	TCACCCCTTTGTTTGACGACA
ELF4	AT2G40080	GGGAGAATCTTGACCGGAA	GACTTGTTGAATCAGTGATCTGTTTC
LUX	AT3G46640	GACGATGATTCTGATGATAAGG	CAGTTTATGCACATCATATGGG
SEN4	AT4G30270	CGTCGATGACACACCCATTAGAG	CATCGGCTTGTTCTTTGGAAAC
